# Middle meningeal artery embolization contraindication when it originates from the ophthalmic artery

**DOI:** 10.1055/s-0044-1786023

**Published:** 2024-04-19

**Authors:** Lívio Pereira de Macêdo, Mayle Gomes Ferreira de Araújo, Pedro Lucas Negromonte Guerra, Marcos Alcino Soares Siqueira Marques, José Laércio Júnior Silva

**Affiliations:** 1 Hospital da Restauração, Departamento de Neurocirurgia, Recife PE, Brazil.; 2 Hospital da Restauração, Departamento de Neurorradiologia Intervencionista, Recife PE, Brazil.; 3 ANGIORAD, Departamento de Radiologia Neurointervencionista, Recife PE, Brazil.; 4 Universidade Federal de Pernambuco, Recife PE, Brazil.

It aims to highlight the importance of anatomical variations of the (MMA) middle meningeal artery's origin in practice for neurointerventionalist. Before proceeding with embolization of the MMA in some pathologies, a critical analysis of the anatomy of these vessels should be done. When MMA originates from the ophthalmic artery, it is a formal contraindication to proceed with the embolization. 1 2 Otherwise, major complications is embolization of the central retinal artery results in blindness. 3

**Figure 1:**
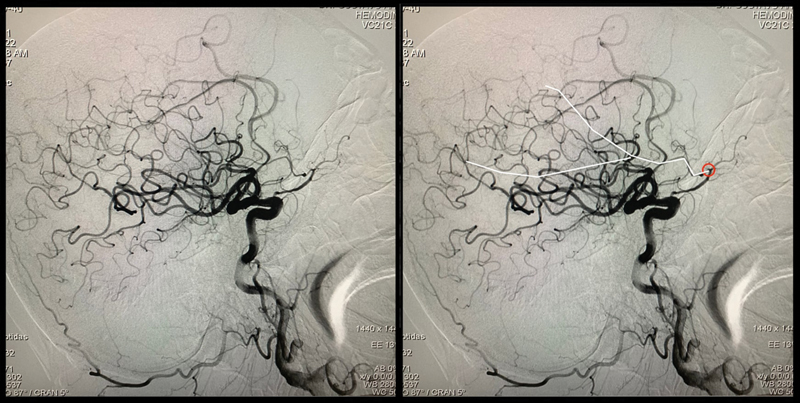
Lateral view of a cerebral angiogram showing a right subdural hematoma and the right middle meningeal artery originates from the ophthalmic artery.

**Figure 2:**
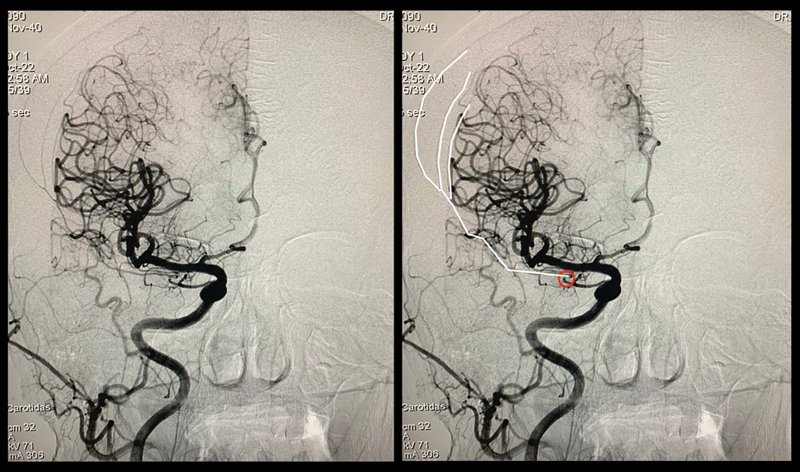
Anteroposterior view of a cerebral angiogram showing a right subdural hematoma and the right middle meningeal artery originates from the ophthalmic artery.

A patient with recurrent chronic subdural hematoma and thrombocytopenia was referred to embolization of the MMA. Angiogram shows MMA originates in the OA, and the procedure was inadvisable ( Figures 1 and 2 ).
